# Statistical methods on detecting differentially expressed genes for RNA-seq data

**DOI:** 10.1186/1752-0509-5-S3-S1

**Published:** 2011-12-23

**Authors:** Zhongxue Chen, Jianzhong Liu, Hon Keung Tony Ng, Saralees Nadarajah, Howard L Kaufman, Jack Y Yang, Youping Deng

**Affiliations:** 1 Biostatistics Epidemiology Research Design Core, Center for Clinical and Translational Sciences, The University of Texas Health Science Center at Houston, Houston, TX 77030, USA; 2 The Chem21 Group Inc, 1780 Wilson Drive, Lake Forest, IL 60045, USA; 3 Department of Statistical Science, Southern Methodist University, Dallas, TX 75275, USA; 4 School of Mathematics, University of Manchester, Manchester, M13 9PL, UK; 5 Rush University Cancer Center, Rush University Medical Center, Chicago, IL 60612, USA; 6 Department of General Surgery and Immunology and Microbiology, Rush University Medical Center, Chicago, IL 60612, USA; 7 Department of Radiation Oncology Massachusetts General Hospital and Harvard Medical School Boston, MA 02114, USA; 8 Department of Internal Medicine and Biochemistry, Rush University Medical Center, Chicago, IL 60612, USA

## Abstract

**Background:**

For RNA-seq data, the aggregated counts of the short reads from the same gene is used to approximate the gene expression level. The count data can be modelled as samples from Poisson distributions with possible different parameters. To detect differentially expressed genes under two situations, statistical methods for detecting the difference of two Poisson means are used. When the expression level of a gene is low, i.e., the number of count is small, it is usually more difficult to detect the mean differences, and therefore statistical methods which are more powerful for low expression level are particularly desirable. In statistical literature, several methods have been proposed to compare two Poisson means (rates). In this paper, we compare these methods by using simulated and real RNA-seq data.

**Results:**

Through simulation study and real data analysis, we find that the Wald test with the data being log-transformed is more powerful than other methods, including the likelihood ratio test, which has similar power as the variance stabilizing transformation test; both are more powerful than the conditional exact test and Fisher exact test.

**Conclusions:**

When the count data in RNA-seq can be reasonably modelled as Poisson distribution, the Wald-Log test is more powerful and should be used to detect the differentially expressed genes.

## Background

Recent advancements in deep sequencing technique enable the ultra-high-throughput sequencing (called the second- or next- generation sequencing) approaches, to be used for transcriptome, including gene expression, analyses [1-4]. RNA-seq produces a count (the total number of short reads which are annotated to positions within a gene or transcript) for each gene expression level. Some researchers have shown that the count data can be quite reasonably modelled as Poisson distribution [1,3]. To detect differentially expressed genes (DEGs) under two conditions (e.g., disease vs. healthy status), some statistical methods have been used. For example, Marioni et al [3] have proposed to use likelihood ratio test (LRT) based on a generalized linear model (GLM) with dependent variable (count) having Poisson distribution to detect DEGs under two conditions without biological replicates. Fisher exact test has also been proposed to detect DEGs for RNA-seq data [5,6]. Some researchers modelled the count data as binomial distribution bin(n,p), where n is the total counts on the same lane, which usually ranges from several millions to tens of millions [7]. However, it is well known that for a binomial distribution bin(n,p) with large n and small p, it can be approximated by Poisson distribution. It is expected that for RNA-seq data methods based on binomial distribution should have similar performance as those methods based on Poisson distribution and therefore are not considered in this paper.

If there are biological replicates, the count data may have larger variances than expected from the Poisson distribution as biological replicates bring extra variances. Under this kind of situations, Poisson distribution with over-dispersed variances or negative binomial distributions are thought to be more appropriate [8,9]. However, due to the high cost, recent studies rarely used biological replicates; we therefore focus on the situations where at most technical replicates are used and Poisson distribution is assumed to be appropriate.

Besides Fisher exact test and LRT from GLM, several other test approaches in statistical literature have been proposed to detect the difference of two Poisson means. For example, under the null hypothesis that X_1 _and X_2 _are both from Poi(*λ*), then given k = X_1_+X_2_, X_1 _has a binomial distribution Bin(k,p), where p = X_1_/k. Therefore the conditional exact test can be used to detect the difference of the two Poisson means. One of the advantages of the GLM is that it can incorporate some covariates that we are interested in. If no covariates are considered due to the small sample size and large number of genes, the LRT can be constructed directly. Similar to LRT, Wald test also has an asymptotical Chi-square distribution with df = 1 [10-12]. A modified Wald test (Wald-Log) with data being first log-transformed has also been proposed for detecting the difference between two Poisson means [11]. Another method called variance stabilizing transformation (VST) was proposed by Huffman [10,13].

In this paper, we first compare the above mentioned methods by using simulations. Since for those methods having the same asymptotic distribution, they are expected to have similar performances for large sample sizes (e.g., Poisson distributions with large means), we focus on simulating Poisson distributions with small or moderate means. We then use a real RNA-seq data to show the performances of those methods.

## Results

### Simulation results

In the simulations, we first assume there are no replicates, i.e., each of the two conditions has only one sample. We assume the data for condition 1 is from a Poisson distribution with fixed mean 5, 10, 15, or 30. For condition 2, we assume the Poisson mean is the same as or greater than that for condition 1. We use the nominal significance level 10^-3 ^to reflect the situation where in a study with a large number of significant variable (genes), a stringent p-value is needed. The estimated size and power are calculated from 10,000 runs for each setting.

**Figure 1:**
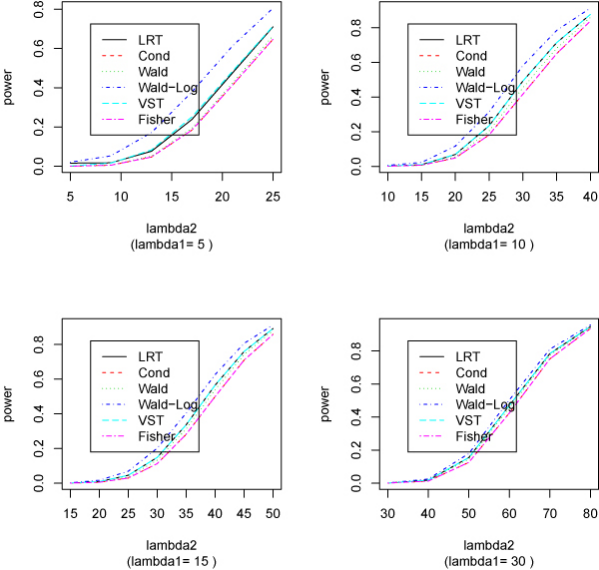
**Estimated power from various methods (no replicates)**. Estimated size and power from each method for detecting the difference of two Poisson means. There are no replicates for each condition. The Poisson means for the first condition are 5, 10, 15, or 30. The second Poisson means are the same as (for size) or larger than (for power) the first ones. The nominal size (significance level) is 10^-3 ^and 10,000 runs are used for each setting.

Table 1 reports the estimated size and power for different situations. Figure 1 plots the estimated size and power for each method from this simulation.

**Table 1:** Estimated size and power (no replicates for each condition).

Lambda1	Lambda2	LRT	Cond	Wald	Wald-Log	VST	Fisher
5	5	0.0128	0.0002	0.0002	0.0185	0.0011	0.0002
	9	0.0199	0.0066	0.0074	0.0522	0.0176	0.0066
	13	0.0842	0.0492	0.0550	0.1737	0.0916	0.0492
	17	0.2484	0.1877	0.1964	0.3976	0.2626	0.1877
	21	0.4903	0.4248	0.4358	0.6269	0.4981	0.4248
	25	0.7146	0.6547	0.6685	0.8077	0.7168	0.6547

10	10	0.0013	0.0004	0.0005	0.0041	0.0013	0.0004
	15	0.0114	0.0082	0.0089	0.0256	0.0124	0.0082
	20	0.0729	0.0504	0.0571	0.1190	0.0737	0.0504
	25	0.2433	0.1863	0.2110	0.3217	0.2435	0.1863
	30	0.4848	0.4111	0.4460	0.5746	0.4848	0.4111
	35	0.7177	0.6625	0.6900	0.7859	0.7177	0.6625
	40	0.8750	0.8414	0.8599	0.9102	0.8750	0.8414

15	15	0.0012	0.0007	0.0009	0.0030	0.0012	0.0007
	20	0.0075	0.0047	0.0052	0.0140	0.0075	0.0047
	25	0.0456	0.0314	0.0377	0.0689	0.0456	0.0314
	30	0.1483	0.1130	0.1305	0.1990	0.1483	0.1130
	35	0.3422	0.2868	0.3167	0.4121	0.3422	0.2868
	40	0.5692	0.5046	0.5459	0.6266	0.5692	0.5046
	45	0.7563	0.7047	0.7393	0.8002	0.7563	0.7047
	50	0.8907	0.8569	0.8796	0.9096	0.8907	0.8569

30	30	0.0011	0.0007	0.0011	0.0019	0.0011	0.0007
	40	0.0180	0.0125	0.0163	0.0224	0.0180	0.0125
	50	0.1464	0.1167	0.1370	0.1704	0.1464	0.1167
	60	0.4776	0.4292	0.4620	0.5109	0.4777	0.4292
	70	0.7840	0.7526	0.7750	0.8145	0.7848	0.7526
	80	0.9421	0.9299	0.9390	0.9514	0.9423	0.9299

Estimated size and power from each method for detecting the difference of two Poisson means. There are no replicates for each condition. The Poisson means for the first condition are 5, 10, 15, or 30. The second Poisson means are the same as (for size) or larger than (for power) the first ones. The nominal size (significance level) is 10^-3 ^and 10,000 runs are used for each setting.

**Figure 2:**
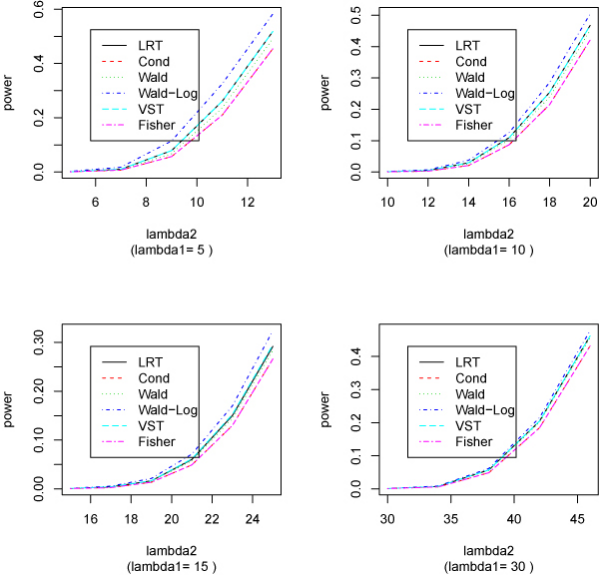
**Estimated power from various methods (with 3 replicates for each condition)**. Estimated size and power from each method for detecting the difference of two Poisson means. There are three replicates for each condition. The Poisson means for the first condition are 5, 10, 15, or 30. The second Poisson means are the same as (for size) or larger than (for power) the first ones. The nominal size (significance level) is 10^-3 ^and 10,000 runs are used for each setting.

In the second simulation, we assume there are three replicates for each condition. Table 2 shows the estimated sizes and powers and these results are plotted in Figure 2.

**Table 2:** Estimated size and power (each condition has three replicates).

Lambda1	Lambda1	LRT	Cond	Wald	Wald-Log	VST	Fisher
5	5	0.0012	0.0009	0.0009	0.0027	0.0012	0.0009
	7	0.0102	0.0062	0.0077	0.0175	0.0103	0.0062
	9	0.0786	0.0542	0.0652	0.1150	0.0786	0.0542
	11	0.2517	0.2008	0.2265	0.3160	0.2517	0.2008
	13	0.5204	0.4581	0.4940	0.5872	0.5204	0.4581

10	10	0.0008	0.0002	0.0006	0.0016	0.0008	0.0002
	12	0.0045	0.0029	0.0042	0.0065	0.0045	0.0029
	14	0.0316	0.0229	0.0279	0.0389	0.0316	0.0229
	16	0.1100	0.0882	0.1019	0.1272	0.1100	0.0882
	18	0.2602	0.2198	0.2454	0.2883	0.2603	0.2198
	20	0.4670	0.4210	0.4515	0.5005	0.4672	0.4210

15	15	0.0007	0.0003	0.0007	0.0007	0.0007	0.0003
	17	0.0034	0.0026	0.0031	0.0049	0.0034	0.0026
	19	0.0181	0.0144	0.0172	0.0221	0.0182	0.0144
	21	0.0612	0.0505	0.0583	0.0712	0.0618	0.0505
	23	0.1442	0.1251	0.1395	0.1648	0.1460	0.1251
	25	0.2850	0.2564	0.2759	0.3125	0.2869	0.2564

30	30	0.0014	0.0010	0.0013	0.0016	0.0014	0.0010
	34	0.0074	0.0057	0.0071	0.0082	0.0074	0.0057
	38	0.0520	0.0457	0.0505	0.0570	0.0520	0.0457
	42	0.2073	0.1846	0.2034	0.2210	0.2073	0.1846
	46	0.4543	0.4270	0.4486	0.4701	0.4543	0.4270

Estimated size and power from each method for detecting the difference of means between two Poisson distributions. There are three replicates for each condition. The Poisson means for the first condition are 5, 10, 15, or 30. The second Poisson means are the same as (for size) or larger than (for power) the first ones. The nominal size (significance level) is 10^-3 ^and 10,000 runs are used for each setting.

From the results of the two simulation studies, we can see that the Wald-Log method is usually more powerful than the LRT, which has similar power as that of VST; both are slightly better than the Wald test, which in turn outperforms Fisher test and conditional binomial test. It is noticeable that both Fisher and conditional tests are exact conditional tests and have almost identical powers.

### Detecting DEGs for a real RNA-seq dataset

**Figure 3:**
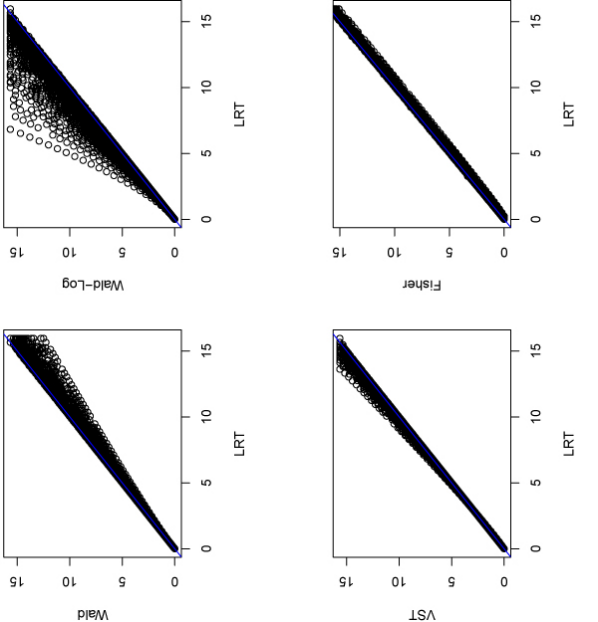
**P-values for the real RNA-seq data from different methods**. Compare p-values (-log10 scale) obtained from other methods with those from LRT for the 15227 genes whose average counts per lane are at least one for each sample.

We also apply these statistical methods to a real RNA-seq data generated by Illumina's sequencing technology [3]. In this dataset, we consider two different samples: kidney and liver both at concentration 3PM, each has 5 lanes (technical replicates). We want to detect DEGs between these two samples. There are 15227 genes with at least 5 counts in total (i.e., on average 1 count on each lane) for both samples. For each of the 15227 genes, we calculate the p-values using various methods mentioned above. Due to the almost identical performance between the Fisher and conditional binomial tests, we choose not to use the latter one in this real data analysis. Figure 3 plots the p-values from other methods compared with the LRT. For many genes, Wald-Log produces smaller p-values than the LRT does.

**Figure 4:**
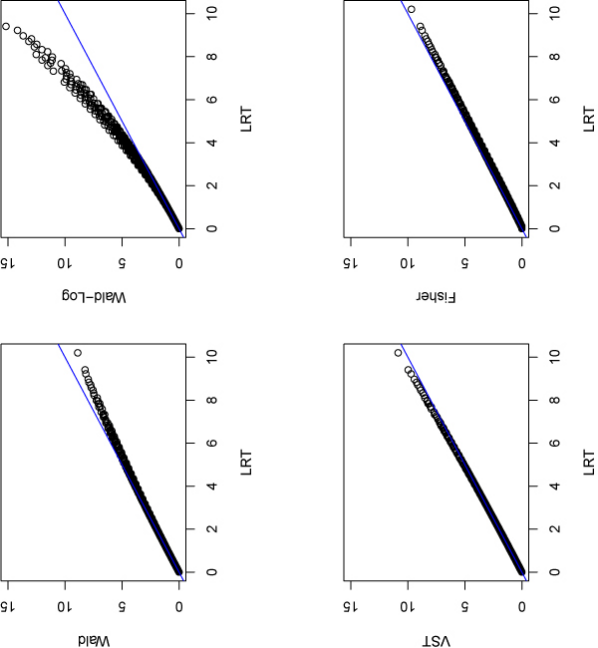
**P-values of the 2657 low espression genes from different methods**. Compare p-values (-log10 scale) obtained from other methods with those from LRT for the 2657 low expression genes whose average counts per lane are between 1 and 10 for each sample.

We are particularly interested in seeing how these statistical methods perform for low expression data. To this purpose, we use the data from genes with average count per lane between 1 and 10. There are 2657 such genes from the data. Figure 4 plots the p-values (on -log10 scale) obtained from other methods compared with those from LRT for those 2657 genes. LRT, Fisher and VST obtain very similar p-values for those genes, while Wald produces larger p-values than LRT for a small portion of those genes. However, for many genes with small p-values from LRT, their p-values obtained by Wald-log are smaller. This indicates the Wald-Log test is more powerful than LRT and other tests for this data.

**Figure 5:**
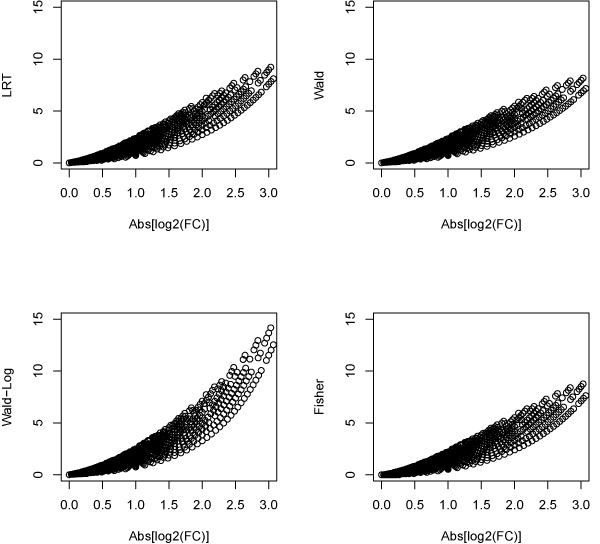
**P-values vs. log2(FC) of the 2657 low espression genes from different methods**. P-value (-log10 scale) obtained by various methods vs. fold change (log2 scale) for the 2657 low expression genes whose average counts per lane are between 1 and 10 for each sample.

To see how statistical significance (p-values) related to biological significance (fold changes), we plot the p-values (on -log10 scale) vs. absolute fold change (on log2 scale) in Figure 5 from different methods for those 2657 genes. It can be seen that the Wald-Log test obtains smaller p-values than other methods when the fold changes are large, indicating this method can detecting more biologically meaningful DEGs than other methods for low expression genes.

**Figure 6:**
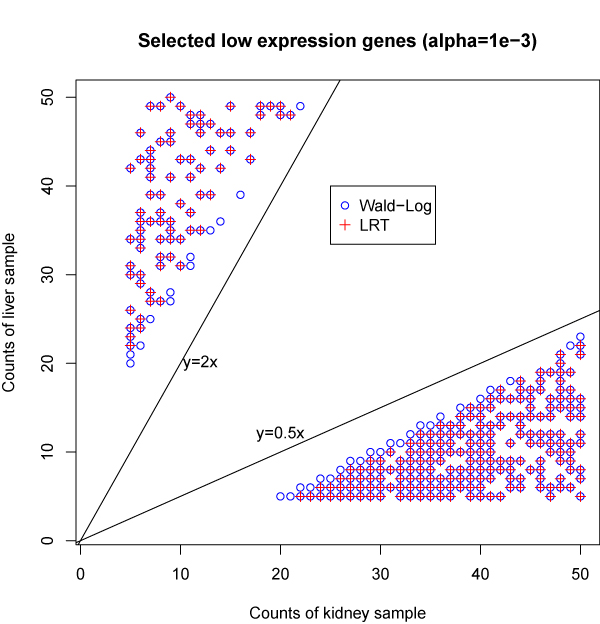
**Counts under two conditions for each of the selected low expression genes**. With significance level 10^-3^, the Wald-Log test selects 581 out of 2657 low expression genes. While the LRT selects 498 of the 581 genes. The count for each selected gene of the two samples is plotted.

Figure 6 plots the counts under two conditions for each low expression genes selected by Wald-Log and LRT at significance level 10^-3^. Of the 581 selected genes by Wald-Log, 498 are also selected by LRT. Each of the selected gene has at least 20 ounts in total for at least one sample (liver or kidney). The fold changes (liver vs. kidney, or kidney vs. liver) of all the 581 selected genes are all at least 2. For those 83 genes selected by Wald-Log but not by LRT, some even have fold changes as large as 4.

Table 3 lists the numbers of DEGs obtained with different cutoff values by various methods. Consistent with what we have observed, the Wald-Log test identifies more DEGs than other methods for low expression genes. This is also true when we apply these statistical tests to all of the 15227 genes.

**Table 3:** Numbers of DEGs of low expression obtained by various methods.

Cutoff	LRT	Cond	Wald	Wald-Log	VST	Fisher
1e-3	498	434	462	581	498	434
1e-4	303	258	270	403	305	260
1e-5	195	166	166	275	201	166
1e-6	113	86	84	209	119	86

Numbers of DEGs selected from the 2657 low expression genes with different cutoff p-values by each method.

## Discussion

Some studies have shown that for RNA-seq data without biological replicates, the count data can be reasonably modelled as Poisson distributed [1,3]. Since it is usually more difficult to detect DEGs with low expression level for RNA-seq data [14], it is desirable to find powerful statistical tests for detecting low expression DEGs. Through simulations and real data, we show that the Wald-Log is more powerful that the commonly used LRT and Fisher exact test. The test statistic of Wald-Log has an asymptotic normal distribution. Like many other test statistics having asymptotic normal or Chi-square distributions, the asymptotic distributional results for the Wald-Log test statistic do not hold when the sample sizes are small (i.e., the Poisson mean is small). However, from our simulations we can see that when the Poisson mean is greater than 10, it can have reasonable sizes even if there are no replicates.

It is interesting to see that the Wald-Log test is also related to the fold changes (see method section for more details); therefore it is also more biologically meaningful. The test statistic of Wald-Log not only uses the fold change (on log scale), but also considers the variances associated with FC. For low expression level, the FC has larger variances, indicating it is more difficult to identify DEGs for low expression genes. This is consistent with the observation from other studies [14,15]: the ability to detect DEGs is strongly associated with the length of gene as longer genes potentially have more aggregated tag counts.

The denominator of the Wald-Log test statistic is: $\sqrt{\left(2+n_{1}∕n_{2}+n_{2}∕n_{1}\right)∕\left(\overset{n_{1}}{\underset{j=1}{\sum }}X_{1j}+\overset{n_{2}}{\underset{j=1}{\sum }}X_{2j}\right)}$, which is the estimated standard error of the fold change (log scale). If we change the square root to: ${\left[\left(2+n_{1}∕n_{2}+n_{2}∕n_{1}\right)∕\left(\overset{n_{1}}{\underset{j=1}{\sum }}X_{1j}+\overset{n_{2}}{\underset{j=1}{\sum }}X_{2j}\right)\right]}^{1∕k}$, then when k = 2, it is the usual Wald-Log test; when *k*→∞, it becomes the fold change criterion as the denominator equals to 1. When the count data has an over-dispersed Poisson distribution, the above estimator underestimates the standard error and we will have inflated type I error rate. Under this situation, we need to derive a new estimator for the standard error; or a Bayesian approach assuming the standard error follows a specific distribution can be built. It should be noticed that for the Bayesian approaches, choosing appropriate prior distributions are critical and may have huge impacts on the results. We may also choose models other than the Poisson model. For example, if there are biological replicates, the count data may have over-dispersed variances, other models such as over-dispersed Poisson distribution or negative binomial distribution can be used [8,9].

## Conclusions

When detecting the difference of two Poisson means, if the Poisson means are large, all the statistical tests mentioned above have very similar power. However, their powers may differ a lot when the Poisson means are small. When the count data in RNA-seq can be reasonably modelled as Poisson distribution, it is desirable to choose a statistical test which outperforms others for low expression genes. Through simulation study and real RNA-seq data analysis, we have shown that the Wald-Log test is more attractive than other methods and should be used to identify DEGs.

## Methods

### Likelihood ratio test (LRT)

Suppose there are n_1 _and n_2 _technical replicates for condition 1 and condition 2, respectively; denote the count of short reads of a gene of the *j^th ^*replicate under condition *i *by x_ij_, where i = 1,2, j = 1,2, ..., n_i_. We also assume random variables X_ij_~Poi(*λ_i_*).

The test statistic of LRT is:

$$T_{LRT}=2\left[\left(\overset{n_{1}}{\underset{j=1}{\sum }}X_{1j}\right)\log\left(\frac{{\hat{\lambda }}_{1}}{{\hat{\lambda }}_{0}}\right)+\left(\overset{n_{2}}{\underset{j=1}{\sum }}X_{2j}\right)\log\left(\frac{{\hat{\lambda }}_{2}}{{\hat{\lambda }}_{0}}\right)+n_{1}\left({\hat{\lambda }}_{0}-{\hat{\lambda }}_{1}\right)+n_{2}\left({\hat{\lambda }}_{0}-{\hat{\lambda }}_{2}\right)\right],$$

where ${\hat{\lambda }}_{0}=\frac{\overset{n_{1}}{\underset{j=1}{\sum }}X_{1j}+\overset{n_{2}}{\underset{j=1}{\sum }}X_{2j}}{n_{1}+n_{2}},\;{\hat{\lambda }}_{1}=\frac{\overset{n_{1}}{\underset{j=1}{\sum }}X_{1j}}{n_{1}},\;{\hat{\lambda }}_{2}=\frac{\overset{n_{2}}{\underset{j=1}{\sum }}X_{2j}}{n_{2}}$.

Under the null hypothesis that the two Poisson means are the same, the test statistic has an asymptotic Chi-square distribution with degree of freedom (df) equals to 1.

LRT has been used by Marioni et al [3] based on a GLM, where they first normalize the data across lanes by a global scale normalization method so that the total counts on each lane are the same after normalization.

### Conditional binomial test

It can be shown that the conditional distribution of the sum of X_1j _given the total sum of X_ij _has a binomial distribution [10,16]. More specifically,

$$\overset{n_{1}}{\underset{j=1}{\sum }}X_{1j}|\overset{n_{1}}{\underset{j=1}{\sum }}X_{1j}+\overset{n_{2}}{\underset{j=1}{\sum }}X_{2j}=k\sim Bin\left(k,p\right),\;\text{where}\;\text{p = }\frac{n_{1}\lambda_{1}}{n_{1}\lambda +n_{2}\lambda_{2}}.$$

Under the null hypothesis, $p_{0}=\frac{n_{1}}{n_{1}+n_{2}}$ and the exact p-value can be calculated from the binomial distribution Bin(n_1_+n_2_, p_0_).

### Wald test

The Wald test statistic is [11]:

$$T_{Wald}=\frac{\sum_{j=1}^{n_{1}}X_{1j}-\frac{n_{1}}{n_{2}}\sum_{j=1}^{n_{2}}X_{2j}}{\sqrt{\frac{n_{1}}{n_{2}}(\sum_{j=1}^{n_{1}}X_{1j}+\sum_{j=1}^{n_{2}}X_{2j}})}.$$

Under the null hypothesis, T_Wald _has an asymptotic standard normal distribution.

### Wald test for logarithm transformed data (Wald-Log)

The Wald test can be also applied to the logarithm transformed count data [11]. We call it Wald-Log test. The test statistic can be derived by using Delta method and has the following form:

$$T_{Wald-Log}=\frac{\log\left[\left(\overset{n_{1}}{\underset{j=1}{\sum }}X_{1j}∕n_{1}\right)∕\left(\overset{n_{2}}{\underset{j=1}{\sum }}X_{2j}∕n_{2}\right)\right]}{\sqrt{\frac{2+n_{1}∕n_{2}+n_{2}∕n_{1}}{\overset{n_{1}}{\underset{j=1}{\sum }}X_{1j}+\overset{n_{2}}{\underset{j=1}{\sum }}X_{2j}}}}=\frac{\log\left(\text{Fold Change}\right)}{\sqrt{\frac{2+n_{1}∕n_{2}+n_{2}∕n_{1}}{\overset{n_{1}}{\underset{j=1}{\sum }}X_{1j}+\overset{n_{2}}{\underset{j=1}{\sum }}X_{2j}}}}.$$

### Variance stabilizing transformation test (VST)

The VST statistic [10,13] has the form:

$$T_{VST}=2[\sqrt{\sum_{j=1}^{n_{1}}X_{1j}+3/8}-\sqrt{(\sum_{j=1}^{n_{2}}X_{2j}+3/8\text{)}n_{1}\text{/}n_{2}}\;]/\sqrt{1+n_{1}/n_{2}}.$$

### Fisher exact test

Fisher exact test is applied to the following 2 by 2 table (Table 4):

**Table 4:** The 2 by 2 table for Fisher test.

Condition 1	$\overset{n_{1}}{\underset{j=1}{\sum }}x_{1j}$	$\overset{n_{1}}{\underset{j=1}{\sum }}m_{1j}-\overset{n_{1}}{\underset{j=1}{\sum }}x_{1j}$
Condition 2	$\overset{n_{2}}{\underset{j=1}{\sum }}x_{2j}$	$\overset{n_{2}}{\underset{j=1}{\sum }}m_{2j}-\overset{n_{2}}{\underset{j=1}{\sum }}x_{2j}$

The 2 by 2 table for Fisher test (where m_ij _is the total number of counts on lane ij).

The exact p-value is then calculated based on the conditional hypergeometric distribution.

## Competing interests

The authors declare that they have no competing interests.

## Authors' contributions

C designed the algorithm, conducted the study, and drafted the manuscript; JL analyzed the data and assisted in programming; YD coordinated and directed the whole project. HKTN, SN, HLK, JYY and YD participated in the analysis and discussion. All authors read and approved the final manuscript.
